# Population-based analysis on predictors for lymph node metastasis in T1 colon cancer

**DOI:** 10.1007/s00464-019-07192-0

**Published:** 2019-10-16

**Authors:** Xin Xu, Chihao Zhang, Xiaochun Ni, Jugang Wu, Chunpeng Pan, Shoulian Wang, Jiwei Yu

**Affiliations:** Department of General Surgery, Shanghai Ninth People’s Hospital, School of Medicine, Shanghai Jiao Tong University, 280 Mohe Road, Shanghai, 201999 China

**Keywords:** Colon cancer, Lymph node metastasis, Independent predictive factors, SEER database

## Abstract

**Background:**

In this study, we aimed to identify independent predictive factors for lymph node metastasis (LNM) in T1 colon cancer.

**Methods:**

Data of 8056 eligible patients were retrospectively collected from the Surveillance, Epidemiology, and End Results (SEER) database during 2004–2012. We performed logistic regression analysis to identify predictive factors for LNM. Both unadjusted and adjusted Cox regression analyses were used to determine the association between LNM and patient survival. Finally, we used competing risks analysis and the cumulative incidence function (CIF) to further confirm the prognostic role of LNM in cancer-specific survival (CSS).

**Results:**

The overall risk of LNM in patients with T1 colon cancer was 12.0% (*N* = 967). Adjusted logistic regression models revealed that mucinous carcinoma [odds ratio (OR) = 2.26, *P* < 0.001], moderately differentiated (OR 1.74, *P* < 0.001), poorly differentiated (OR 5.16, *P* < 0.001), and undifferentiated carcinoma (OR 3.01, *P* = 0.003); older age (OR 0.66, *P* < 0.001 for age 65–79 years, OR 0.44, *P* < 0.001 for age over 80 years); and carcinoma located in the ascending colon (OR 0.77, *P* = 0.018) and sigmoid colon (OR 1.24, *P* = 0.014) were independent predictive factors for LNM. Adjusted Cox regression analysis showed that positive lymph node involvement was significantly associated with CSS [hazard ratio (HR) = 3.02, *P* < 0.001], which was further robustly confirmed using a competing risks model and the CIF.

**Conclusions:**

This population-based study showed that mucinous carcinoma, tumor grade, age, and primary tumor location were independent predictive factors for LNM in T1 colon cancer. The risk of LNM should be carefully evaluated in patients with T1 colon cancer, before clinical management.

Colorectal cancer is among the leading causes of cancer-related mortality in both western countries and China [1, 2]. Colorectal cancer is mainly divided into colon cancer and rectal cancer based on primary tumor location, with colon cancer accounting for approximately 70% of colorectal cancers [1, 3]. Early colon cancer refers to carcinoma with invasion limited to the submucosa [4, 5], which can be designated T1N_X_M0 based on the TNM classification system.

T1 colon cancer is heterogeneous in its clinical presence and prognostic outcome [4]. Generally, the long-term survival of patients with stage I colorectal cancer is excellent after radical resection [6]. The risk of lymph node metastasis (LNM) has been reported to range between 8 and 15% [6–8] in T1 colorectal cancer. The probability of lymph node involvement is considered in the clinical management of colon cancer because lymph node status substantially affects patient prognosis [9]. On the one hand, inadequate removal of positive regional lymph nodes would increase local recurrence and cause poor prognosis. On the other hand, extensive surgical resection that is unnecessary would lead to low quality of life and postoperative morbidity.

Advanced endoscopic techniques have become established therapeutic approaches in patients with T1 colon cancer who are carefully selected and evaluated [8, 10]. As LNM occurs in approximately 10% of all T1 colorectal cancers [7, 11], unnecessary additional surgical resection might be avoided after initial endoscopic resection and careful evaluation to eliminate any possible risk factors, including LNM. For this proportion of patients, unnecessary surgery would cause anastomotic leakage and bowel dysfunction but would yield no survival benefit [12]. However, for patients with a high risk of LNM, surgical resection is required to decrease the local recurrence rate and subsequently increase survival. Therefore, to establish a proper therapeutic strategy and minimize the local recurrence rate, patients with a high risk of LNM should be identified.

To this end, we aimed to determine the predictors for LNM in T1 colon cancer using data of eligible patients from the Surveillance, Epidemiology, and End Results (SEER) database in the present study.

## Materials and methods

### Data source and patient selection

The National Cancer Institute-based SEER database covers approximately 28% of all cancer cases and includes 18 population-based cancer registries in the USA [13]. SEER is also one of the largest publicly accessible databases globally and is updated annually. In this study, relevant data were retrieved from the SEER database. This study was approved by the institutional ethical review board of Shanghai Ninth People’s Hospital, School of Medicine, Shanghai Jiao Tong University.

A total of 8056 eligible patients were enrolled between 2004 and 2012, according to the following inclusion criteria: (1) patients age 18 years or over; (2) a pathological diagnosis of T1 adenocarcinoma or mucinous adenocarcinoma of the colon; (3) at least 12 lymph nodes sampled; and (4) undergoing active follow-up. Patients were eliminated if they had in situ cancer, underwent preoperative radiotherapy, or experienced another primary malignancy.

Data on patient demographics (age, sex, year at diagnosis, ethnicity, and marital status) and tumor characteristics [tumor size, histology, carcinoembryonic antigen (CEA) level, tumor grade, primary tumor site, number of resected lymph nodes, and postoperative radiation] were retrieved from the SEER database and subsequently analyzed.

Overall survival (OS) was defined as time from the date of diagnosis until death for any reason, or the last follow-up. Cancer-specific survival (CSS) was defined as time from the date of diagnosis until death attributed to colon cancer.

### Statistical analysis

Chi-square or Fisher’s exact tests were used to compare categorical variables. An unadjusted logistic regression model, adjusted logistic regression model, and backward logistic regression model were used to identify and confirm risk factors for positive lymph node involvement. Odd ratios (ORs) and 95% confidence intervals (CIs) were determined. A Cox regression model was used to identify independent prognostic factors for OS and CSS. In addition, OS and CSS curves were generated using the Kaplan–Meier method, with a log-rank test to determine statistical significance. Finally, a competing risks model was established and the cumulative incidence function (CIF) was estimated. SPSS version 13.0 (SPSS Inc., Chicago, IL, USA) and R software for Windows version R-3.4.3 (The R Foundation for Statistical Computing, Vienna, Austria) were used for statistical analysis. A two-sided *P* value < 0.05 was considered to indicate statistical significance.

## Results

### Baseline characteristics

The patient selection process is shown in Table 1. Of the data of 161,589 patients diagnosed with colon cancer who underwent surgical resection during 2004–2012 from the SEER database, 8056 eligible patients were finally included in the present analysis. A total of 3924 male and 4132 female patients were included. The median number of lymph nodes sampled was 17 [interquartile range (IQR): 14–22]. The overall risk of LNM in patients with T1 colon cancer was 12.0% (*N* = 967). The median follow-up was 68 months (ranging from 47 to 94 months). At the end of follow-up, 6650 (82.55%) patients were still alive. The cancer-specific mortality rate was 9.41% (*N* = 91) and 3.26% (*N* = 231) in patients with and without LNM, respectively. Other detailed clinicopathological information is shown in Table 2.

**Table 1:**
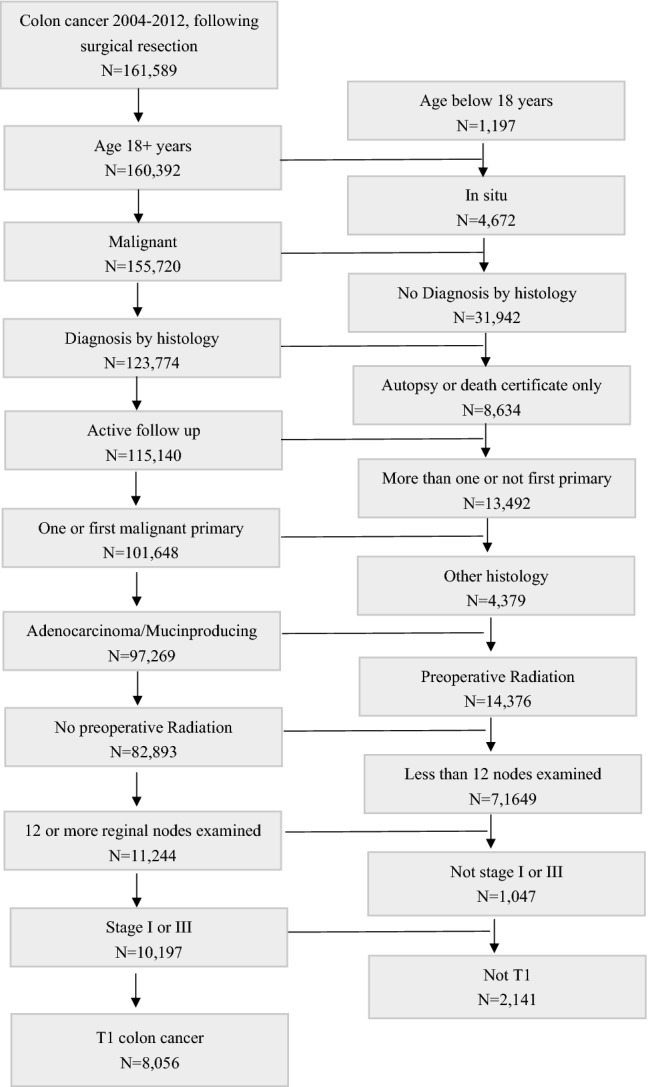
Flowchart of patient selection

**Table 2:** Clinicopathological characteristics of the selected patients

	Total *N *= 8056	*N *= 967	N0 *N *= 7089	*P*
Tumor size (cm)				0.015
< 1	1442 (17.9)	169 (17.48)	1273 (17.96)	
1–1.9	1736 (21.55)	231 (23.89)	1505 (21.23)	
2–2.9	1160 (14.4)	137 (14.17)	1023 (14.43)	
3+	1482 (18.4)	200 (20.68)	1282 (18.08)	
Not stated	2236 (27.76)	230 (23.78)	2006 (28.3)	
Histology				< 0.001
Adenocarcinoma	7835 (97.26)	920 (95.14)	6915 (97.55)	
Mucinous carcinoma	221 (2.74)	47 (4.86)	174 (2.45)	
CEA*				< 0.001
Positive	457 (5.67)	75 (7.76)	382 (5.39)	
Negative	3123 (38.77)	439 (45.4)	2684 (37.86)	
Borderline/unknown	4476 (55.56)	453 (46.85)	4023 (56.75)	
Grade				< 0.001
Well-differentiated	1605 (19.92)	116 (12.0)	1489 (21)	
Moderately differentiated	5054 (62.74)	625 (64.63)	4429 (62.48)	
Poorly differentiated	563 (6.99)	162 (16.75)	401 (5.66)	
Undifferentiated	55 (0.68)	10 (1.03)	45 (0.63)	
Unknown	779 (9.67)	54 (5.58)	725 (10.23)	
Year				0.112
2004–2006	1680 (20.85)	216 (22.34)	1464 (20.65)	
2007–2009	2998 (37.21)	331 (34.23)	2667 (37.62)	
2010–2012	3378 (41.93)	420 (43.43)	2958 (41.73)	
Age (years)				< 0.001
Up to 49	731 (9.07)	114 (11.79)	617 (8.7)	
50–64	3101 (38.49)	455 (47.05)	2646 (37.33)	
65–79	3162 (39.25)	324 (33.51)	2838 (40.03)	
80+	1062 (13.18)	74 (7.65)	988 (13.94)	
Gender				0.257
Male	3924 (48.71)	454 (46.95)	3470 (48.95)	
Female	4132 (51.29)	513 (53.05)	3619 (51.05)	
Race				0.321
Black	988 (12.26)	117 (12.1)	871 (12.29)	
White	6371 (79.08)	753 (77.87)	5618 (79.25)	
Others	647 (8.03)	88 (9.1)	559 (7.89)	
Unknown	50 (0.62)	9 (0.93)	41 (0.58)	
Marital status				0.007
Married	4865 (60.39)	626 (64.74)	4239 (59.8)	
Single/widowed	2042 (25.35)	228 (23.58)	1814 (25.59)	
Other/unknown	1149 (14.26)	113 (11.69)	1036 (14.61)	
Primary site				< 0.001
Cecum	1781 (22.11)	204 (21.1)	1577 (22.25)	
Ascending colon	1989 (24.69)	181 (18.72)	1808 (25.5)	
Hepatic flexure	399 (4.95)	42 (4.34)	357 (5.04)	
Transverse colon	615 (7.63)	61 (6.31)	554 (7.81)	
Splenic flexure	157 (1.95)	13 (1.34)	144 (2.03)	
Descending colon	410 (5.09)	49 (5.07)	361 (5.09)	
Sigmoid colon	2705 (33.58)	417 (43.12)	2288 (32.28)	
Total lymph nodes Median (IQR*)	17 (14–22)	17 (14–22)	17 (14–22)	0.375
Radiation				< 0.001
None	8043 (99.84)	959 (99.17)	7084 (99.93)	
Postoperative	13 (0.16)	8 (0.83)	5 (0.07)	
Cause of death				< 0.001
Alive	6650 (82.55)	786 (81.28)	5864 (82.72)	
Dead from cancer	322 (4.00)	91 (9.41)	231 (3.26)	
Dead not from cancer	1084 (13.46)	90 (9.31)	994 (14.02)	
Follow-up time (months)	68 (47–94)	66 (45–94)	66 (45–94)	0.176

*CEA carcinoembryonic antigen, *IQR* interquartile range

### Risk factors of lymph node metastasis

Unadjusted and adjusted multivariate logistic regression analyses were used to determine the risk factors for LNM. As a result, mucinous carcinoma, tumor grade, age, and primary tumor location were robustly confirmed as significant predictive factors for LNM (Table 3). Patients with mucinous carcinoma had significantly higher risks of LNM. Compared with patients who had well-differentiated colon cancer, those with moderately differentiated, poorly differentiated, and even undifferentiated carcinoma were at higher risk of LNM. In terms of age, a decreasing LNM risk was detected in older patients (age 65–79 years and age over 80 years). Of note, carcinoma located in the ascending colon and sigmoid colon was significantly associated with lower LNM risk, as compared with carcinoma located in the cecum.

**Table 3:** Logistic regression analysis of the risk factors for lymph node metastasis in T1 colon cancer

	Unadjusted logistic regression		Adjusted logistic regression		Adjusted selection from adjusted logistic regression	
	OR* (95% CI*)	*P*	OR (95% CI)	*P*	OR (95% CI)	*P*
Tumor size (cm)						
< 1	Reference					
1–1.9	1.16 (0.94–1.43)	0.180				
2–2.9	1.01 (0.79–1.28)	0.943				
3+	1.18 (0.94–1.46)	0.149				
Not stated	0.86 (0.70–1.07)	0.173				
Histology						
Adenocarcinoma	Reference		Reference		Reference	
Mucinous carcinoma	2.03 (1.45–2.80)	< 0.001	2.26 (1.61–3.21)	< 0.001	2.30 (1.61–3.21)	< 0.001
CEA*						
Positive	Reference		Reference		Reference	
Negative	0.83 (0.64–1.10)	0.181	0.76 (0.58–0.99)	0.047	0.76 (0.58–1.00)	0.047
Borderline/unknown	0.57 (0.44–0.75)	< 0.001	0.56 (0.43–0.74)	< 0.001	0.56 (0.43–0.74)	< 0.001
Grade						
Well-differentiated	Reference		Reference		Reference	
Moderately differentiated	1.81 (1.48–2.24)	< 0.001	1.74 (1.42–2.15)	< 0.001	1.74 (1.42–2.15)	< 0.001
Poorly differentiated	5.19 (3.99–6.75)	< 0.001	5.16 (3.96–6.75)	< 0.001	5.16 (3.96–6.75)	< 0.001
Undifferentiated	2.85 (1.33–5.58)	0.004	3.01 (1.39–5.97)	0.003	3.01 (1.39–5.97)	0.003
Unknown	0.96 (0.68–1.33)	0.793	0.56 (0.64–1.26)	0.571	0.91 (0.64–1.26)	0.571
Year						
2004–2006	Reference					
2007–2009	0.84 (0.70–1.01)	0.064				
2010–2012	0.96 (0.81–1.15)	0.669				
Age (years)						
Up to 49	Reference		Reference		Reference	
50–64	0.93 (0.75–1.17)	0.528	0.99 (0.79–1.26)	0.970	0.99 (0.79–1.26)	0.970
65–79	0.62 (0.49–0.78)	< 0.001	0.66 (0.52–0.84)	< 0.001	0.66 (0.52–0.84)	< 0.001
80+	0.41 (0.30–0.55)	< 0.001	0.44 (0.32–0.60)	< 0.001	0.44 (0.32–0.60)	< 0.001
Gender						
Male	Reference					
Female	1.08 (0.95–1.24)	0.243				
Race						
Black	Reference					
White	0.99 (0.81–1.23)	0.983				
Others	1.17 (0.87–1.57)	0.294				
Marital status						
Married	Reference		Reference		Reference	
Single/widowed	0.85 (0.72–0.99)	0.050	0.98 (0.82–1.15)	0.781	0.98 (0.82–1.15)	0.781
Other/unknown	0.74 (0.60–0.91)	0.005	0.73 (0.59–0.91)	0.005	0.73 (0.59–0.91)	0.005
Primary site						
Cecum	Reference		Reference		Reference	
Ascending colon	0.77 (0.63–0.96)	0.017	0.77 (0.62–0.96)	0.018	0.77 (0.62–0.96)	0.018
Hepatic flexure	0.91 (0.63–1.28)	0.597	0.91 (0.63–1.29)	0.592	0.91 (0.63–1.29)	0.592
Transverse colon	0.85 (0.62–1.14)	0.296	0.81 (0.59–1.10)	0.184	0.81 (0.59–1.10)	0.184
Splenic flexure	0.70 (0.37–1.21)	0.229	0.63 (0.33–1.11)	0.134	0.63 (0.33–1.11)	0.134
Descending colon	1.05 (0.75–1.45)	0.777	0.92 (0.64–1.28)	0.612	0.92 (0.64–1.28)	0.612
Sigmoid colon	1.41 (1.18–1.69)	< 0.001	1.26 (1.05–1.53)	0.014	1.26 (1.05–1.53)	0.014

*OR odd ratio, *95% CI* 95% confidence intervals, *CEA* carcinoembryonic antigen

### Lymph node metastasis and patient survival

**Fig. 1:**
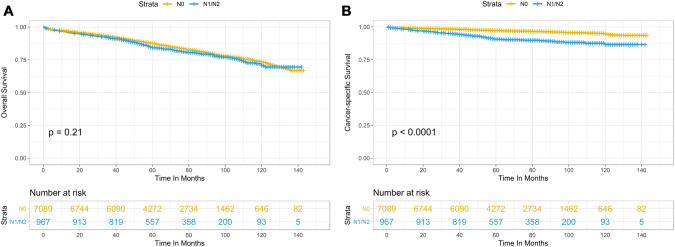
Effect of lymph node metastasis on overall survival (**A**) and cancer-specific survival (**B**) in T1 colon cancer

We further evaluated the association between LNM and patient survival. Unadjusted and adjusted multivariate Cox regression models persistently showed that tumor size, CEA level, age, and marital status were significant prognostic factors for OS in patients with T1 colon cancer (Table 4). Similarly, lymph node status, tumor size, CEA level, tumor grade, year at diagnosis, age, and marital status had significant prognostic value for CSS in patients with T1 colon carcinoma (Table 5). Interestingly, positive lymph node involvement was significantly associated with CSS [hazard ratio (HR) = 3.02 (2.34–3.89), *P* < 0.001 in adjusted analysis] but not with OS [HR = 1.11 (0.95–1.29), *P* = 0.21 in unadjusted analysis]. To further investigate the prognostic significance of LNM, patients were categorized into two groups according to their lymph node status. Kaplan–Meier curves showed no statistical significance of OS between the two groups (*P* = 0.21) (Fig. 1A), whereas the CSS rate was significantly lower in the lymph node positive group than that in the lymph node negative group (*P* < 0.0001) (Fig. 1B).

**Table 4:** Cox regression analysis of prognostic factors for overall survival in T1 colon cancer

	Unadjusted		Adjusted		Variable selection	
	HR* (95% CI*)	*P*	HR (95% CI)	*P*	HR (95% CI)	*P*
N stage						
N0	Reference					
N1/N2	1.11 (0.95–1.29)	0.21				
Tumor size (cm)						
< 1	Reference		Reference		Reference	
1–1.9	1.16 (0.98–1.39)	0.091	1.08 (0.91–1.29)	0.405	1.09 (0.91–1.30)	0.348
2–2.9	1.41 (1.17–1.69)	< 0.001	1.21 (1.01–1.46)	0.043	1.23 (1.02–1.48)	0.029
3+	1.45 (1.22–1.73)	< 0.001	1.29 (1.08–1.53)	0.005	1.30 (1.09–1.54)	0.004
Not stated	0.86 (0.72–1.02)	0.086	0.93 (0.78–1.11)	0.433	0.93 (0.78–1.11)	0.448
Histology						
Adenocarcinoma	Reference		Reference			
Mucinous carcinoma	1.47 (1.14–1.91)	0.003	1.12 (0.86–1.46)	0.391		
CEA*						
Positive	Reference		Reference		Reference	
Negative	0.40 (0.33–0.48)	< 0.001	0.50 (0.41–0.60)	< 0.001	0.49 (0.41–0.60)	< 0.001
Borderline/unknown	0.51 (0.43–0.61)	< 0.001	0.61 (0.51–0.73)	< 0.001	0.61 (0.51–0.72)	< 0.001
Grade						
Well-differentiated	Reference		Reference			
Moderately differentiated	1.04 (0.91–1.19)	0.600	1.05 (0.91–1.20)	0.502		
Poorly differentiated	1.10 (0.88–1.37)	0.422	1.12 (0.89–1.40)	0.340		
Undifferentiated	2.31 (1.43–3.72)	< 0.001	2.02 (1.25–3.26)	0.004		
Unknown	0.90 (0.73–1.11)	0.320	1.05 (0.85–1.30)	0.666		
Year						
2004–2006	Reference					
2007–2009	1.02 (0.90–1.16)	0.745				
2010–2012	0.86 (0.74–1.01)	0.063				
Age (years)						
Up to 49	Reference		Reference		Reference	
50–64	1.84 (1.25–2.71)	0.002	1.92 (1.30–2.82)	0.001	1.91 (1.30–2.82)	0.001
65–79	5.39 (3.72–7.82)	< 0.001	5.33 (3.67–7.76)	< 0.001	5.37 (3.70–7.80)	< 0.001
80+	17.44 (12.00–25.36)	< 0.001	15.68 (10.72–22.91)	< 0.001	15.82 (10.86–23.04)	< 0.001
Gender						
Male	Reference					
Female	0.99 (0.89–1.10)	0.827				
Race						
Black	Reference		Reference		Reference	
White	1.00 (0.85–1.17)	0.954	0.78 (0.67–0.92)	0.004	0.78 (0.66–0.92)	0.003
Others	0.56 (0.42–0.74)	< 0.001	0.50 (0.38–0.66)	< 0.001	0.50 (0.38–0.66)	< 0.001
Marital status						
Married	Reference		Reference		Reference	
Single/widowed	2.10 (1.88–2.35)	< 0.001	1.38 (1.23–1.55)	< 0.001	1.38 (1.23–1.55)	< 0.001
Other/unknown	1.05 (0.89–1.25)	0.562	1.02 (0.86–1.21)	0.823	1.02 (0.86–1.21)	0.839
Primary site						
Cecum	Reference		Reference			
Ascending colon	0.99 (0.86–1.13)	0.866	1.07 (0.93–1.23)	0.369		
Hepatic flexure	0.87 (0.68–1.11)	0.264	0.95 (0.73–1.21)	0.658		
Transverse colon	0.73 (0.58–0.91)	0.006	0.91 (0.73–1.14)	0.405		
Splenic flexure	0.72 (0.48–1.07)	0.106	1.05 (0.70–1.58)	0.803		
Descending colon	0.64 (0.49–0.85)	0.002	0.96 (0.73–1.28)	0.802		
Sigmoid colon	0.58 (0.50–0.67)	< 0.001	1.01 (0.87–1.17)	0.883		

*HR hazard ratio, *95% CI* 95% confidence intervals, *CEA* carcinoembryonic antigen

**Table 5:** Cox regression analysis of prognostic factors for cancer-specific survival in T1 colon cancer

	Unadjusted		Adjusted		Variable selection	
	HR* (95% CI*)	*P*	HR (95% CI)	*P*	HR (95% CI)	*P*
N stage						
N0	Reference		Reference		Reference	
N1/N2	2.95 (2.31–3.76)	< 0.001	3.02 (2.34–3.89)	< 0.001	3.00 (2.33–3.87)	< 0.001
Tumor size (cm)						
< 1	Reference		Reference		Reference	
1–1.9	1.22 (0.84–1.79)	0.296	1.09 (0.74–1.60)	0.661	1.09 (0.74–1.60)	0.649
2–2.9	1.44 (0.97–2.15)	0.073	1.21 (0.81–1.80)	0.364	1.21 (0.81–1.80)	0.341
3+	1.90 (1.33–2.73)	< 0.001	1.57 (1.09–2.26)	0.015	1.57 (1.09–2.26)	0.014
Not stated	0.88 (0.60–1.29)	0.504	0.93 (0.63–1.37)	0.702	0.93 (0.63–1.37)	0.709
Histology						
Adenocarcinoma	Reference		Reference		Reference	
Mucinous carcinoma	2.11 (1.33–3.36)	0.002	1.45 (0.90–2.33)	0.124	1.45 (0.90–2.33)	0.114
CEA*						
Positive	Reference		Reference		Reference	
Negative	0.33 (0.23–0.47)	< 0.001	0.40 (0.28–0.58)	< 0.001	0.40 (0.28–0.58)	< 0.001
Borderline/unknown	0.35 (0.25–0.50)	< 0.001	0.45 (0.32–0.63)	< 0.001	0.45 (0.32–0.64)	< 0.001
Grade						
Well-differentiated	Reference		Reference		Reference	
Moderately differentiated	1.06 (0.79–1.42)	0.697	1.03 (0.77–1.38)	0.834	1.02 (0.76–1.37)	0.874
Poorly differentiated	1.92 (1.29–2.87)	0.001	1.49 (0.99–2.24)	0.057	1.48 (0.98–2.23)	0.060
Undifferentiated	3.43 (1.48–7.93)	0.004	3.03 (1.30–7.05)	0.010	2.99 (1.29–6.96)	0.011
Unknown	0.77 (0.48–1.24)	0.285	0.88 (0.54–1.44)	0.604	0.88 (0.54–1.43)	0.599
Year						
2004–2006	Reference		Reference		Reference	
2007–2009	0.86 (0.66–1.12)	0.274	0.91 (0.69–1.19)	0.474	0.91 (0.70–1.19)	0.488
2010–2012	0.63 (0.46–0.87)	0.004	0.67 (0.49–0.92)	0.014	0.68 (0.50–0.93)	0.016
Age (years)						
Up to 49	Reference		Reference		Reference	
50–64	0.96 (0.59–1.54)	0.855	1.07 (0.66–1.74)	0.778	1.07 (0.66–1.74)	0.774
65–79	1.58 (0.99–2.50)	0.052	1.63 (1.02–2.60)	0.040	1.65 (1.04–2.62)	0.033
80+	3.53 (2.19–5.70)	< 0.001	3.33 (2.03–5.45)	< 0.001	3.37 (2.08–5.47)	< 0.001
Gender						
Male	Reference					
Female	0.96 (0.77–1.19)	0.694				
Race						
Black	Reference					
White	1.00 (0.85–1.17)	0.954				
Others	0.56 (0.42–0.74)	< 0.001				
Marital status						
Married	Reference		Reference		Reference	
Single/widowed	2.00 (1.58–2.53)	< 0.001	1.62 (1.27–2.06)	< 0.001	1.62 (1.27–2.06)	< 0.001
Other/unknown	1.02 (0.72–1.46)	0.905	1.01 (0.70–1.44)	0.971	1.01 (0.70–1.44)	0.967
Primary site						
Cecum	Reference		Reference			
Ascending colon	1.02 (0.76–1.37)	0.897	1.19 (0.88–1.60)	0.263		
Hepatic flexure	0.82 (0.48–1.41)	0.479	0.91 (0.53–1.56)	0.725		
Transverse colon	0.77 (0.48–1.24)	0.284	1.01 (0.63–1.62)	0.982		
Splenic flexure	0.13 (0.02–0.96)	0.045	0.20 (0.03–1.42)	0.107		
Descending colon	0.73 (0.41–1.29)	0.280	1.03 (0.58–1.83)	0.916		
Sigmoid colon	0.73 (0.54–0.98)	0.038	1.04 (0.76–1.42)	0.810		

*HR hazard ratio, *95% CI* 95% confidence intervals, *CEA* carcinoembryonic antigen

### Competing risk analysis

The prognostic outcomes of cancer patients are influenced by both oncological factors and non-oncological factors. Therefore, cancer patients might die from other causes before cancer-specific death occurs [14].

**Fig. 2:**
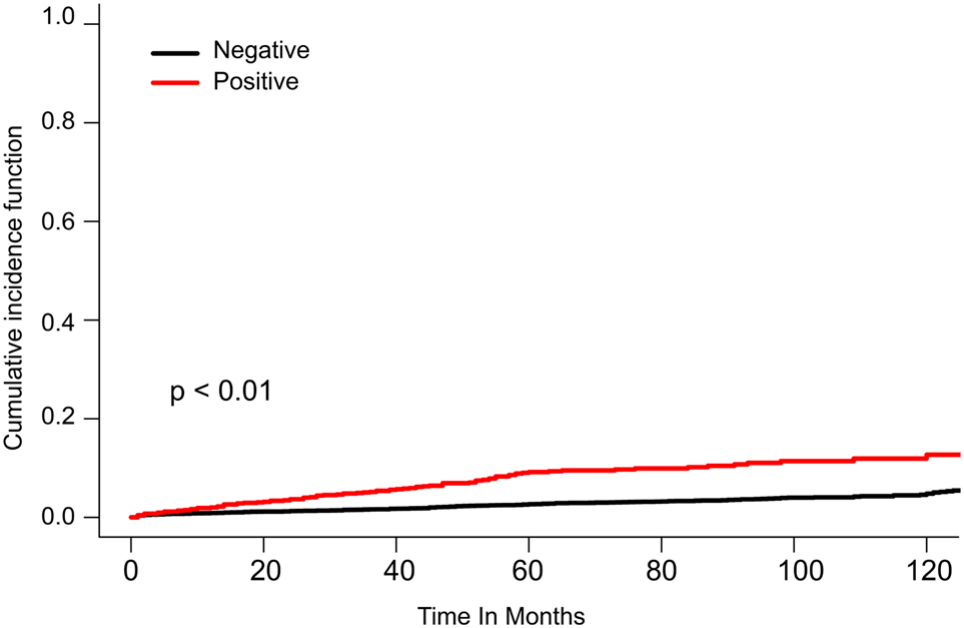
Cumulative incidence function for cancer-specific death. Black curve indicates cancer-specific death without lymph node metastasis; red curve indicates cancer-specific death with lymph node metastasis in T1 colon cancer (Color figure online)

For accurate determination of the prognostic role of LNM in T1 colon cancer, a competing risks model was used, which directly links the effects of risk factors with cause-specific cumulative incidence of death [15]. As a result, LNM [subdistribution hazard ratio (SHR) = 2.96, *P* < 0.001], tumor size > 3.0 cm (SHR = 1.50, *P* = 0.026), negative CEA level (SHR = 0.45, *P* < 0.001), poorly differentiated (SHR = 1.60, *P* < 0.031) or undifferentiated (SHR = 2.91, *P* = 0.022) carcinoma, diagnosis during 2010–2012 (SHR = 0.60, *P* = 0.001), older age (SHR = 1.61, *P* = 0.048 for age 65–79 years; SHR = 3.01, *P* < 0.001 for age over 80 years), white ethnicity (SHR = 0.57, *P* < 0.001), and single/widowed marital status were all significant prognostic factors for T1 colon cancer (Table 6). In addition, the CIF was used to evaluate the probability of cancer-specific mortality and death from other causes [16]. As shown in Fig. 2, the cancer-specific death rate was significantly higher in patients with LNM (shown as a red curve) than in patients without LNM (shown as a black curve).

**Table 6:** Competing risks analysis for cancer-specific death

	SHR* (95% CI*)	*P*
N stage		
N0	Reference	
N1/N2	2.96 (2.30–3.82)	< 0.001
Tumor size (cm)		
< 1	Reference	
1–1.9	1.09 (0.75–1.59)	0.670
2–2.9	1.17 (0.78–1.74)	0.461
3+	1.50 (1.03–2.19)	0.026
Not stated	0.93 (0.63–1.37)	0.720
Histology		
Adenocarcinoma	Reference	
Mucinous carcinoma	1.46 (0.91–2.36)	0.130
CEA*		
Positive	Reference	
Negative	0.45 (0.31–0.64)	< 0.001
Borderline/unknown	0.48 (0.34–0.68)	< 0.001
Grade		
Well-differentiated	Reference	
Moderately differentiated	1.08 (0.80–1.45)	0.730
Poorly differentiated	1.60 (1.06–2.42)	0.031
Undifferentiated	2.91 (1.19–7.15)	0.022
Unknown	0.90 (0.55–1.49)	0.601
Year		
2004–2006	Reference	
2007–2009	0.84 (0.65–1.10)	0.180
2010–2012	0.60 (0.44–0.82)	0.001
Age (years)		
Up to 49	Reference	
50–64	1.06 (0.65–1.72)	0.841
65–79	1.61 (1.00–2.58)	0.048
80+	3.01 (1.82–4.98)	<0.001
Race		
Black	Reference	
White	0.57 (0.43–0.76)	< 0.001
Others	0.48 (0.28–0.81)	0.007
Marital status		
Married	Reference	
Single/widowed	1.45 (1.13–1.87)	0.003
Other/unknown	0.96 (0.67–1.38)	0.860
Primary site		
Cecum	Reference	
Ascending colon	1.21 (0.89–1.65)	0.230
Hepatic flexure	0.96 (0.56–1.65)	0.881
Transverse colon	1.05 (0.66–1.69)	0.832
Splenic flexure	0.20 (0.03–1.39)	0.102
Descending colon	1.09 (0.61–1.92)	0.781
Sigmoid colon	1.12 (0.81–1.55)	0.490

*SHR subdistribution hazard ratio, *95% CI* 95% confidence intervals, *CEA* carcinoembryonic antigen

## Discussion

With great advances in endoscopic techniques, endoscopic resection is advantageous for low-risk submucosal colon cancer, which dramatically decreases postoperative morbidities, increases quality of life, and gives rise to relatively good long-term clinical outcomes comparable to those of radical surgical resection. However, the indications of endoscopic resection in T1 colon cancer should be cautiously managed. In a retrospective study including 428 patients with T1 colorectal cancer [17], the authors indicated that the conventional indications for endoscopic treatment should not be expanded, mainly owing to the risk of LNM. Therefore, accurate identification of the predictors for LNM risk is crucial to distinguishing patients with low risk of LNM who can thus be treated using endoscopic resection, with oncological outcomes comparable to those of radical resection.

In this population-based study, we investigated the predictors for LNM in T1 colon cancer. Mucinous carcinoma, tumor grade, age, and primary tumor location were significant predictors for LNM. Mucinous carcinoma is a relatively rare pathological type of colorectal cancer, accounting for approximately 10–15% of all colorectal cancer cases [18]. As a distinct subtype, mucinous carcinoma has been reported to be associated with higher risks of lymph node involvement in stage I and II colorectal cancer [19, 20]. Our population-based analysis consistently revealed that patients with mucinous carcinoma of the colon had a higher risk of LNM. Not surprisingly, tumor grade was significantly predictive for lymph node involvement. Of note, poorly differentiated carcinoma increased LNM risk by more than 5 times, in comparison with well-differentiated carcinoma, in all three logistic regression models. Consistent with previous findings in T1 rectal cancer [21], in the present study, we identified older age as a significant negative predictor for LNM. Compared with patients age up to 49 years, the risk of LNM in patients age 65–79 years and more than 80 years dropped to approximately 0.65 and 0.44, respectively (both *P* < 0.001). It has been reported that lymph node yield declines with age in patients with colorectal cancer, with mean lymph node yield reduced by 1 for every 7-year increase in age overall [22].

Primary tumor location has long been reported to have an impact on the risk of LNM in colorectal cancer [4, 23]. The LNM risk in T1 rectal carcinoma has been revealed to be as high as 15% [4, 5, 24], dropping to 8% in the left colon and 3% in the right colon [4]. Here, we report similar observations, which suggests that carcinoma of the ascending colon is a significant negative predictor for the risk of LNM, whereas sigmoid colon cancer significantly increases the LNM risk. The differing LNM risks according to different primary tumor locations might be owing to intrinsic genetic differences [4, 25]. Unlike other studies concerning rectal cancer [21], we found that tumor size was not a predictive factor for the risk of LNM in T1 colon cancer. Consistent with our findings, Okabe et al. also demonstrated an insignificant association between tumor size and LNM risk in T1 adenocarcinoma of the colon and rectum [4]. Therefore, it remains controversial whether primary tumor size is a predictive factor for the risk of LNM in T1 colorectal cancer, a question that deserves further investigation.

During the patient selection process, patients without an adequate number of resected lymph nodes were excluded. The cutoff value for the number of sampled lymph nodes was set to 12, according to the general consensus that at least 12 lymph nodes are required for accurate pathological judgement [26]. In this population-based analysis, LNM was detected in 12.0% (967 out of 8056) of patients with T1 colon cancer, which was slightly higher than the proportion in other studies [4, 27]. It is feasible that the lymph node positive rate increases with an increased number of sampled lymph nodes. In this study, only patients with more than 12 resected lymph nodes were enrolled, which might give rise to a slightly higher LNM rate in our study.

In survival analysis, LNM was a significant prognostic factor for CSS but not for OS. Patients with T1 colon cancer generally have good prognosis. In this study, the cancer-specific death rate and noncancer-specific death rate were 3.26% and 14.02%, respectively, for patients without LNM (Table 2). However, these rates were comparable to those in patients with LNM (9.41% for cancer-specific death and 9.31% for noncancer-specific death). The above observations robustly indicate the importance of lymph node status in determining oncological outcome in T1 colon cancer.

Owing to relatively long survival in patients with T1 colon cancer, long-term patient survival is influenced by other noncancer risks. That is to say, a considerable proportion of patients might die from causes other than cancer-related causes [15, 28, 29]. Therefore, to accurately illustrate the prognostic role of lymph node status in T1 colon cancer, we constructed a competing risks model and estimated the CIF. LNM was revealed as a definite risk factor for prognosis in patients with T1 colon cancer.

In the present population-based analysis, our conclusions are based on real-world outcomes. With a median follow-up of 68 months among 8056 eligible participants, we report these convincing findings with a high degree of statistical power. Nevertheless, certain limitations must be acknowledged. The limited availability of data from the SEER database is the main drawback. Factors including submucosal invasion depth, tumor budding, and lymphovascular invasion might also affect the likelihood of LNM, which were not assessed in our study. In terms of primary tumor location, ascending colon and sigmoid colon carcinomas are significant predictors for lymph node involvement; however, we failed to reveal any association of the hepatic flexure, transverse colon, splenic flexure, and descending colon with the risk of LNM. The relatively small sample of these tumor locations might be the cause.

In conclusion, the overall LNM rate is approximately 12.0% for T1 colon cancer. Mucinous carcinoma, tumor grade, age, and primary tumor location are significant predictors for LNM in patients with T1 colon cancer. Moreover, positive lymph node involvement is a significant prognostic factor for CSS. Thus, careful preoperative assessment of lymph node status is essential in clinical decision making, to achieve better long-term outcomes.
